# Supplementary material for: Association between oral microbiome and seven types of cancers in East Asian population: a two-sample Mendelian randomization analysis
Source: Front Mol Biosci. 2023 Nov 21;10:1327893. doi: 10.3389/fmolb.2023.1327893 (PMC10702768; doi:10.3389/fmolb.2023.1327893)
Supplement: Supplementary file 1 [file DataSheet1.ZIP › Supplementary Materials/MR plots of saliva/saliva═╝/ovarian cancer/pheno.782_to_ovarian cancer_forest.pdf]

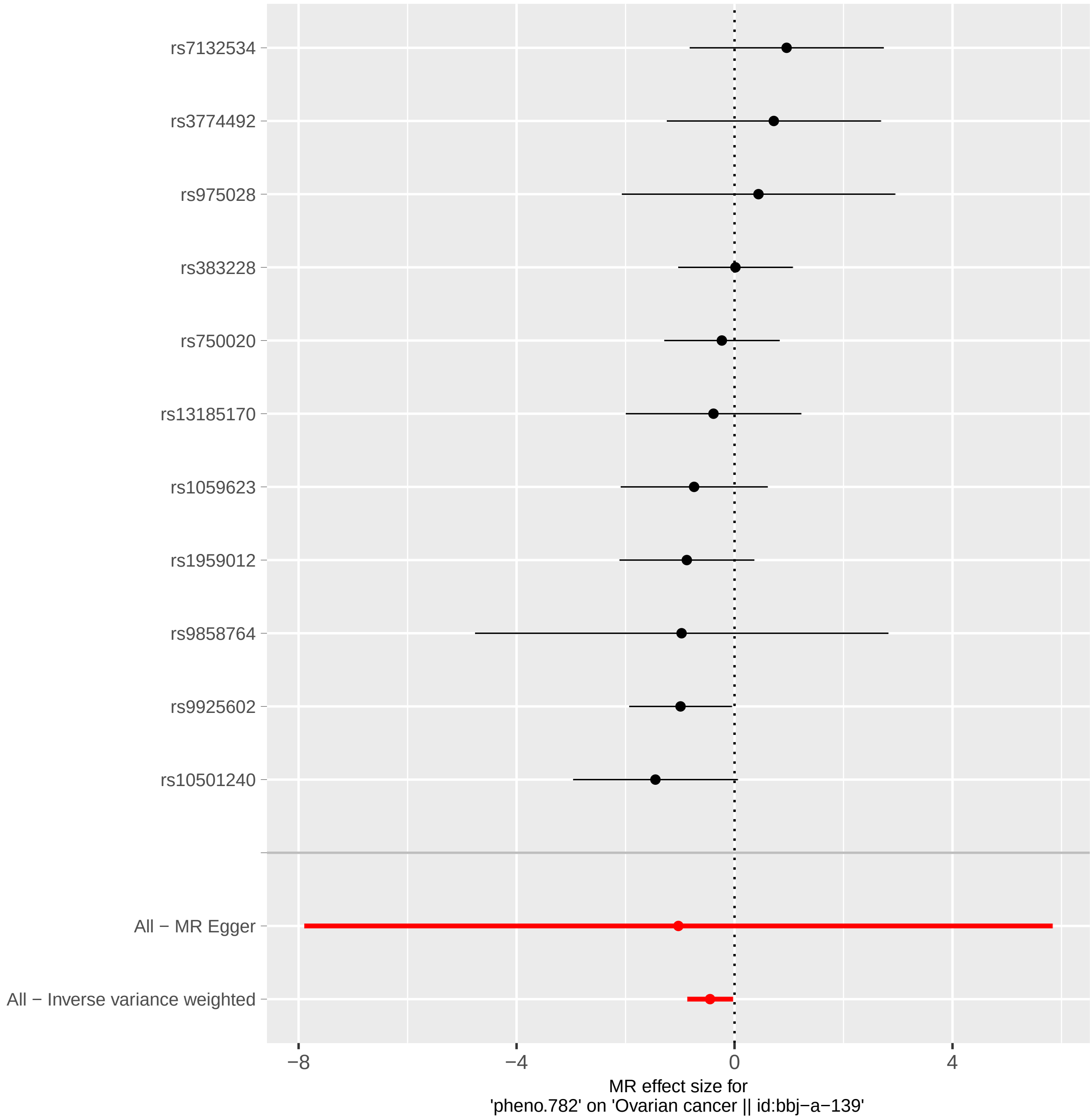

rs7132534
rs3774492
rs975028
rs383228
rs750020
rs13185170
rs1059623
rs1959012
rs9858764
rs9925602
rs10501240
All − MR Egger
All − Inverse variance weighted
−8
−4
0
4
MR effect size for
'pheno.782' on 'Ovarian cancer || id:bbj−a−139'
